# Knowledge distillation approach for skin cancer classification on lightweight deep learning model

**DOI:** 10.1049/htl2.12120

**Published:** 2025-01-15

**Authors:** Suman Saha, Md. Moniruzzaman Hemal, Md. Zunead Abedin Eidmum, Muhammad Firoz Mridha

**Affiliations:** ^1^ Department of IoT and Robotics Engineering Bangabandhu Sheikh Mujibur Rahman Digital University, Bangladesh Gazipur Bangladesh; ^2^ Department of Computer Science American International University‐Bangladesh Dhaka Bangladesh

**Keywords:** convolutional neural nets, health care, learning (artificial intelligence)

## Abstract

Over the past decade, there has been a global increase in the incidence of skin cancers. Skin cancer has serious consequences if left untreated, potentially leading to more advanced cancer stages. In recent years, deep learning based convolutional neural network have emerged as powerful tools for skin cancer detection. Generally, deep learning approaches are computationally expensive and require large storage space. Therefore, deploying such a large complex model on resource‐constrained devices is challenging. An ultra‐light and accurate deep learning model is highly desirable for better inference time and memory in low‐power‐consuming devices. Knowledge distillation is an approach for transferring knowledge from a large network to a small network. This small network is easily compatible with resource‐constrained embedded devices while maintaining accuracy. The main aim of this study is to develop a deep learning‐based lightweight network based on knowledge distillation that identifies the presence of skin cancer. Here, different training strategies are implemented for the modified benchmark (Phase 1) and custom‐made model (Phase 2) and demonstrated various distillation configurations on two datasets: HAM10000 and ISIC2019. In Phase 1, the student model using knowledge distillation achieved accuracies ranging from 88.69% to 93.24% for HAM10000 and from 82.14% to 84.13% on ISIC2019. In Phase 2, the accuracies ranged from 88.63% to 88.89% on HAM10000 and from 81.39% to 83.42% on ISIC2019. These results highlight the effectiveness of knowledge distillation in improving the classification performance across diverse datasets and enabling the student model to approach the performance of the teacher model. In addition, the distilled student model can be easily deployed on resource‐constrained devices for automated skin cancer detection due to its lower computational complexity.

## Graphical abstract

Deploying complex convolutional neural network models for skin cancer detection on resource‐constrained devices is challenging. This study presents an ultra‐light deep learning model using knowledge distillation, improving classification accuracy while ensuring efficient inference and low power consumption. This approach enhances accessibility for healthcare providers in resource‐limited settings.

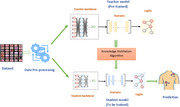

**Abbreviations**
AUC: area under the curve
ACO: ant colony optimization
BCC: basal cell carcinoma
BKL: keratosis‐like lesions (KL)
CNN: convolutional neural network
CRF: conditional random field
DF: dermatofibroma
FCN: fully convolutional network
KD: knowledge distillation
KNN: k‐nearest neighbour
MLP: multi‐layer perceptron
O‐SVM: optimized support vector machine
SCC: squamous cell carcinoma
IRF: interferon regulatory factor

## 1 INTRODUCTION

Skin cancer is one of the most dangerous forms of cancer, caused by unrepaired DNA damage that initiates mutations in the skin, leading the skin cells to reproduce rapidly and form malignant tumours [1]. It affects a large number of individuals worldwide, particularly the fair‐skinned population [2]. There are three primary categories of skin cancer: basal cell carcinoma (BCC), squamous cell carcinoma (SCC), and melanoma. Among these, basal cell carcinoma and squamous cell carcinoma are commonly referred to as “non‐melanoma skin cancer.” On the other hand, melanoma is not as common as basal cell or squamous cell carcinomas but is the most dangerous. It can spread quickly to other parts of the body and can be fatal if not treated early [3]. Generally, skin cancer spreads to other organs of the body slowly, and the increasing rates of skin cancer and expensive healthcare maintenance demand that the disease be identified and diagnosed in the early stage which plays an important role in controlling skin cancers and their successful treatment [4]. Owing to the rapid advancement of artificial intelligence (AI) technology, deep learning has been widely used to identify and diagnose various skin diseases in medical imaging. Using deep learning‐based approaches in skin cancer detection can improve the outcomes for individuals with skin cancer by increasing the accuracy, speed, and accessibility of diagnosis and treatment. Among the various deep learning algorithms, convolutional neural network (CNN) based architectures are the most popular and successful in detecting skin cancer and have achieved excellent performance. Because CNN can extract features in classification, increasing number of studies underway to create advanced deep CNN networks to enhance the diagnostic performance of skin cancer detection and classification [5]. With further improvements in deep learning algorithms, more novel networks have also been suggested and are being utilized for this task. Considering the popularity of deep learning, there are still many problems to overcome and options to pursue in the future.

CNN architectures have shown great success in skin cancer detection owing to their ability to extract meaningful features from images. CNN can learn and analyze image patterns at different levels of complexity, making them highly effective in identifying the abnormal features of skin lesions. Previous research studies have shown that existing CNN architectures usually focuses on pursuing high prediction accuracies, ignoring the constraint of computing resources on mobile devices or less powerful embedded devices. Table 1 shows the number of parameters, memory, and flops for different CNN architectures. It is apparent that running resource‐intensive CNN architectures on a simple processor of resource‐constrained devices can be challenging due to limited memory and processing power. Examples of powerful processors that can perform GFLOPS include Intel Xeon Phi, NVIDIA Tesla V100, AMD Ryzen Threadripper 3990X, etc. Usually, ultra‐light models with a few thousand parameters can be used in resource‐constrained devices, but the trade‐off is that they may not provide the same level of accuracy as more prominent and more complex models. Researchers are constantly exploring new techniques to design more efficient models that can achieve higher accuracy with fewer parameters and fewer computational resources.

**TABLE 1:** Number of parameters, memory and flops for different CNN architectures.

Model	Parameters	Parameters memory	Feature memory	FLOPs (GF)
AlexNet	60 M	229.4 MB	56.9 MB	2
Inception‐v3	25 M	91 MB	89 MB	6
Inception‐v4	42 M	162 MB	40.1 MB	13
Xception	22 M	84 MB	21 MB	8
Resnet‐101	44.5 M	170 MB	155 MB	8
Resnet‐152	60 M	230 MB	219 MB	11
Densenet‐161	20 M	110 MB	235 MB	8
Densenet‐201	20 M	77 MB	19 MB	8
VGG‐16	138 M	528 MB	58 MB	16
VGG‐19	138 M	548 MB	63 MB	20

Several techniques are available to reduce model size and computational complexity, making it possible to run these models on resource‐constrained devices. These techniques include model compression, transfer learning, knowledge distillation, etc. Among them, the knowledge distillation proposed by Hinton [6] based deep neural network has gained popularity due to its effectiveness in reducing the size and complexity of models without sacrificing too much accuracy and is utilized as an efficient tool to enhance the adaptability of ultralight models under low resources. Knowledge distillation is a method of transferring knowledge from an extensive network to a smaller network. We believe that knowledge distillation is promising for improving the performance of smaller and, more efficient models for detecting skin lesions, which could have important implications for developing more accessible and affordable diagnostic tools for skin cancer. By doing this, the smaller model can achieve comparable performance to the larger model without sacrificing too much accuracy while using fewer resources. This study aims to develop an efficient model architecture for skin cancer detection using knowledge distillation techniques. The major contributions of this study are outlined below.

- A smaller, more computationally efficient model that can classify skin cancer images with comparable accuracy to a larger, more complex model.
- Improve skin disease classification performance when dealing with medical imaging devices in real‐world scenarios.
- An empirical study of the effectiveness of the architecture was conducted through rigorous simulation of different publicly available datasets.

The remainder of this paper is organized as follows: Section 2 presents a comprehensive review of the literature related to skin cancer detection using CNN architectures, including recent advancements, challenges, and limitations. Section 3 focuses on knowledge distillation approaches and provides a detailed discussion of the theoretical background of this technique. Section 4 outlines the methodology used to apply knowledge distillation to the skin cancer classification problem. Section 5 presents the experimental results obtained in the simulation environment. Finally, Section 6 concludes the study.

## 2 LITERATURE REVIEW

In this section, we discuss several deep CNN architecture designed to recognize skin lesions and categorize them into various forms of skin cancer.

### 2.1 Skin cancer classification based on VGG16 & VGG‐19 CNN architecture

An overview of the research utilizing neural networks to categorize skin cancers was included in this study [7]. The authors compared different neural network models, including multi‐layer perceptron (MLP), a custom CNN, and the pre‐trained VGG‐16 model. Experimental results showed that pre‐trained models and custom CNN were more effective in learning skin cancer traits than MLP. For categories such as Basal Cell Carcinoma and Vascular, the pre‐trained VGG‐16 model achieved the highest validation accuracy among all the classes. Although, training CNN models took longer than MLP models because of their complex architecture, they had faster testing times and did not require retraining when deployed. The study in [8] addressed skin lesion analysis by proposing two deep learning‐based techniques for assessing dermoscopic images of skin malignancies. The first technique focuses on lesion segmentation from the surrounding skin by using a fully convolutional–deconvolutional architecture. The second technique involves the classification of skin cancers using both a simple convolutional neural network and a transfer learning‐based VGG‐16 architecture. The proposed models were trained and evaluated on standard benchmark datasets from the International Skin Imaging Collaboration (ISIC) 2017 challenge. The recommended strategies showed promising results with an average Jaccard index of 0.507 for lesion segmentation using a fully convolutional deconvolutional architecture. In [9], Salian et al. presented a skin lesion categorization system that utilized pre‐trained CNN architectures along with a custom CNN model. The proposed method enhances tagged images, extracts features, and predicts the skin lesions. Two datasets, PH2 and HAM10000, were used for evaluation. The experimental results showed that the custom CNN model outperformed the pre‐trained models on both data sets. The authors of [10] explored various classification algorithms, including Support Vector Machines (SVM), Decision Trees, Linear Discriminant Analysis, K‐nearest neighbour (KNN), and a transfer learning approach using the VGG‐16 CNN architecture for the detection of skin cancer. From the experiment, it was found that the K‐nearest neighbour technique combined with the VGG‐16 CNN model achieved the highest accuracy of 99%. Linear Discriminant Analysis, Decision Trees, and KNN classifiers all performed well with a classification accuracy higher than 99%. On the other hand, the authors in [11] focused on classifying three distinct skin lesions: dermatofibroma (DF), keratosis‐like lesions (KL), and basal cell carcinoma (BCC) using a CNN model based on VGG19 and Transfer Learning. The study used the HAM10000 dataset, selecting three specific skin cancer types while addressing dataset imbalance through data augmentation techniques. The results showed high accuracy and minimal loss. The network demonstrated stability and prevented overfitting.

### 2.2 Skin cancer classification based on ResNet CNN architecture

Authors in [12] introduced the optimal ResNet model over the ISIC 2018 dataset. Five ResNet models with depths of 50, 40, 25, 10, and 7 were tested to determine the best performance. The experimental result show that the ResNet 50 model without data augmentation achieved the highest validation accuracy (0.83) and F1 score (0.46). The study [13] utilized the ISIC dataset containing 25,331 dermatoscopic colour images of skin lesions categorized into nine classes. This research focused on improving image separation through preprocessing techniques and proposed the use of a residual network (ResNet) architecture for effective skin lesion diagnosis. The proposed ResNet architecture, specifically ResNet‐34, outperformed simpler networks and achieved an accuracy of 0.92 for skin lesion classification. In [14], the authors focused on the early detection of skin cancer by classifying the images as benign or malignant. Here, the authors employed two deep learning architectures, ResNet‐101 and Inception‐v3, to assess classification performance. The Inception‐v3 model outperformed the ResNet‐101 model in classification performance, achieving accuracy rates of 87.42% and 84.09%, respectively. The study also demonstrated that deep learning approaches, represented by these two algorithms, can be effective in accurately identifying and diagnosing skin cancer. The study in [15] presented an early‐stage deep learning approach for identifying, segmenting, and classifying skin lesions. It utilizes various models such as tinyYOLOv2, 3D‐semantic segmentation, optimized feature selection with ant colony optimization (ACO) and classifiers such as optimized support vector machine (O‐SVM). The experimental result showed that the proposed method achieved an accuracy of 97.8% when tested on the ISBI 2017‐2018 and 2019 datasets, which include diverse skin cancer images. This technique showed promise for the early detection and diagnosis of skin lesions.

### 2.3 Skin cancer classification based on DenseNet architecture

In [16], the authors introduced a novel approach for automated skin cancer diagnosis by combining lesion segmentation and classification. The proposed architecture consists of two stages: the first stage utilizes an encoder decoder fully convolutional network (FCN) for learning skin lesion details, and the second stage employs a DenseNet framework for classification. The conditional random field (CRF) module was integrated for lesion border localization and contour refinement. The model achieved impressive results on the HAM10000 dataset, with 98% accuracy, 98.5% recall, and 99% area under the curve (AUC). In [17], a fine‐grained classification approach was proposed to identify light skin cancers. The model comprises two feature extraction modules and a feature discrimination network. The fusion of these approaches enhances the performance of the model while maintaining a low number of parameters. The proposed method outperformed state‐of‐the‐art deep learning‐based methods on the ISBI 2016 skin lesion analysis dataset. The application of deep learning to hierarchical skin abnormality identification was explored in [18]. Here, the authors investigated the significance of colour normalization, lesion segmentation, and transfer learning techniques in developing a deep learning‐based diagnostic system for ISIC 2017‐ISBI. On the other hand, to assist healthcare professionals in increasing the efficiency of their analysis, research [19] recommended a DenseNet model to enhance the efficiency of melanoma detection in skin lesion images. The model was trained and tested on the ISIC2020 dataset, outperforming previous deep learning methods with an AUC score of 0.925. The study details the model training process, including dataset utilization, pre‐trained DenseNet201 model from ImageNet for feature extraction, ADAM optimizer, and learning rate.

### 2.4 Skin cancer classification using knowledge distillation

Knowledge distillation, is a widely adopted concept that creates efficiency in model size and, consequently, increases inference speed, without loss of accuracy. This has enjoyed considerable success in other tasks such as melanoma detection. In [20], the authors introduced a lightweight student model, the so‐called distilled student network (DSNet), trained using knowledge distillation from the teacher model, namely ResNet‐50. The approach by the authors had an accuracy of 91.7% with only 0.26 million parameters, which is much more compact than the teacher model of 42.5 million parameters. They further report faster inference times of 2.57 s compared to 14.55 s of the pre‐trained models such as MobileNet, VGG‐16, and EfficientNet‐B0. The reduction in model size and runtime makes knowledge distillation a feasible solution for the real‐time detection of melanoma on memory‐constrained devices. Going one step further from the knowledge distillation approach, another paper [21] discussed the advantage of having multiple teacher models to train a smaller student network on the classification task of skin cancer detection. They fine‐tuned two teacher models‐ResNet50 and DenseNet161‐to accuracies of 98.32% and 98.80%, respectively. However, both of these models are very computationally expensive and hard to deploy on edge devices. For comparison, they also provided a student model called TinyStudent with only 0.35 million parameters, reaching an accuracy of 85.45% and 85.00% for ResNet50 and DenseNet161, respectively. While such a reduction in accuracy may occur, TinyStudent is 82 times smaller compared to DenseNet161 and hence could be feasible on resource‐constrained devices. Knowledge from multiple teacher models further improves the performance, whereby the ensemble of models reaches accuracies of 87.74% and 88.00%. This study emphasizes the need to strike a balance between model accuracy and real‐world constraints on deployment to edge devices. Mutual learning is another strategy for improving the performance of student models. Two major limitations brought about by traditional knowledge distillation are: (i) the passivity of the student model while learning from the teacher, and (ii) the inability of the teacher model to incorporate the relation between samples while training, which were the basis for the proposal in [22]. This paper proposes the relationship‐aware mutual learning method: under mutual bidirectional knowledge distillation, two identical student models were adopted; each student was equipped with a sample relationship module to improve the mutual learning process for better sample relationship capturing. Extensive experiments were conducted by the authors, who evaluated the impact of mutual learning and relationship modules on model performance and substantial improvements were observed. For instance, using MobileNetV2 for both the teacher and student models increased the accuracy from 87.4%, where no mutual learning was used, to 88.3% upon the incorporation of mutual learning and relationship modules. Similarly, the BACC increased from 84.5% to 85.3%, whereas the mAP increased from 0.765 to 0.801. Based on the above discussion, it can be said that CNN‐based architectures are well‐suited for skin cancer detection. However, this approach typically necessitates the analysis of large numbers of high‐resolution images, which can be computationally expensive and memory‐intensive. Existing studies have primarily focused on improving the accuracy, overlooking the development of a framework for a simple setup that can be embedded in resource‐constrained portable devices. To address this issue, we applied the knowledge distillation method which transforms complicated models into simple models. This study aims to develop a knowledge‐distillation framework to enhance the auto‐recognition of skin diseases based on lightweight deep‐learning models that can be easily deployed on resource‐constrained IoT devices. The proposed architecture improves the skin cancer prediction capability of the lightweight model while maintaining its runtime efficiency.

## 3 KNOWLEDGE DISTILLATION

**FIGURE 1:**
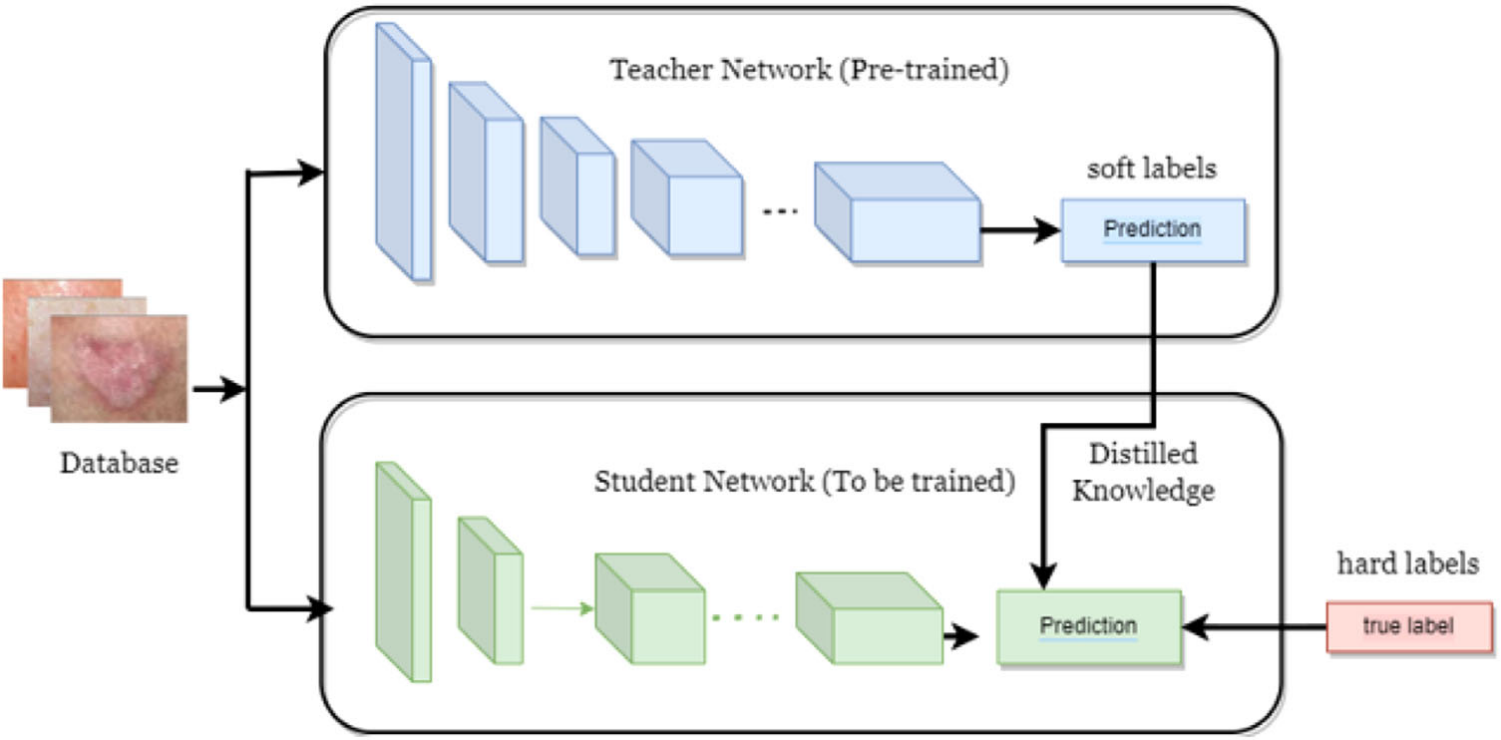
Knowledge distillation approach.

The basic idea of knowledge distillation, also known as the teacher student approach, employs a complex network as a teacher model to teach a smaller network as a student model as shown in Figure 1. This means the information of the teacher is being distilled into the student. On the other hand, the student model trained by knowledge distillation absorbs more knowledge than during conventional training. As a result, the student model can perform similarly to the teacher model, but with low computational mobile devices.

### 3.1 Components of the knowledge distillation

The major components of the knowledge distillation include the following.

#### 3.1.1 Knowledge

There are three different forms of knowledge within a teacher model in knowledge distillation: Response‐based, feature‐based, and relation‐based knowledge [23]. Response‐based knowledge focuses on imitating the final prediction of the teacher model. The goal of feature‐based knowledge is to teach the student model to understand the identical feature activations as the teacher model by reducing the distinction between the feature activations of the teacher and student models. On the other hand, relation‐based knowledge explores the associations between various layers or data models.

#### 3.1.2 Distillation schemes

The learning strategies for knowledge distillation can be categorized into three types: Offline distillation, online distillation, and self‐distillation. In offline distillation, the knowledge of a pre‐trained teacher model is transferred to the student model. In online distillation, the goal is to collaboratively teach an ensemble of multiple student models and distil knowledge from each other. On the other hand, self‐distillation connects multiple attention modules and external classifiers at various model depths and distils knowledge from the deepest architectures to the shallower ones.

#### 3.1.3 Architecture

In knowledge distillation, teacher student architecture is very important to form knowledge transfer. The complexity of deep neural networks mostly arises from two proportions: depth and width. Generally, it is necessary to disseminate knowledge from deeper to shallower networks. Therefore, student networks can be one of the following forms: (i) A simplified structure of a teacher model with fewer layers and fewer channels in each layer; (ii) a quantized structure of a teacher model in which the configuration of the network is conserved; (iii) a simple structure with the necessary and efficient functions; and (iv) a simple structure with an optimized network architecture.

#### 3.1.4 Algorithms

Different types of distillation algorithms have been proposed to simplify the process of transferring knowledge in complex scenarios. The recently introduced knowledge distillation approaches for knowledge transfer include adversarial distillation, multi‐teacher distillation, cross‐modal distillation, graph‐based distillation, attention‐based distillation, data‐free distillation, quantized distillation, lifelong distillation, NAS‐based distillation, etc.

### 3.2 Knowledge distillation with Hinton algorithm

The knowledge distillation algorithm introduced by Hinton et al. [6] uses two loss functions to accomplish the distillation. The first loss function compares the student model's predictions (softmax distribution) with the one‐hot encoded hard labels of the data. The purpose of the second loss function was to compare the softmax distributions (soft labels) of the student and teacher models. A well‐trained teacher model typically generates a high pseudo‐probability for the correct answer, which is similar to one‐hot encoding. However, it also produces non‐zero pseudo‐probabilities for incorrect classes. Unlike others, from these incorrect likelihoods, the student model can learn a lot of useful information from the false soft labels, as shown by Hinton et al [6]. Consider training a child to classify fruits. When knowing about apples, it may be useful to understand how identical the attributes of an apple are to those of pear fruit and how dissimilar they are from an orange. The soft target loss function performs a very similar effect by guiding the learner to the complete spectrum of class likelihoods. Therefore, the student model as a learner may require much less data to acquire all the knowledge of the teacher model by specifying classes that are overlooked from the training data. When conducting knowledge distillation using Hinton's algorithm, it is necessary to loosen the softmax distributions from the teacher and the student wherein the pseudo likelihoods are distributed evenly. The final output layer of a deep model is the softmax layer which converts the logits $z_{i}$ for *i*‐th class into the probability $p_{i}$ belongs to the *i*‐th class by the following equation:

$$p_{i}=\text{softmax}\left(z_{i}\right)=\frac{exp(z_{i})}{\sum_{j}exp\left(z_{j}\right)}$$

Usually the hard targets are known as the one‐hot label which is a category vector with one entry being 1 and the rest being 0, such as [0, 0, 1, 0]. On the other hand, the soft targets are somewhat probabilistic distributions of different categories, such as [0.15, 0.2, 0.6, 0.05]. The prediction of the soft target by the teacher model includes dark knowledge and can be utilized as a coach to transfer information from the teacher network to the small network. Furthermore, a temperature factor *T* is introduced to superintend the significance of each soft target as follows.

$$p_{i}=\text{softmax}\left(z_{i}\right)=\frac{exp(z_{i}/T)}{\sum_{j}exp\left(z_{j}/T\right)}$$

Here, a high‐temperature value has a softer probability distribution over the classes. Especially, when *T* →∞, all classes belong to the same likelihood. On the other hand, for *T* → 0, the soft targets convert to the hard targets. However, the Hinton‐loss function is defined as follows.

$$L_{\text{KD}}=\alpha E_{h}\left(y_{p},y_{h}\right)+\beta E_{s}\left(y_{p}^{\prime },y_{s}^{\prime }\right)$$

where, $L_{\text{KD}}$: Hinton‐loss function, $E_{h}$: cross‐entropy for the hard labels, $E_{s}$: cross‐entropy for the soft labels, $y_{p}$: prediction obtained by student model, $y_{s}$: prediction of the soft labels by the teacher, $y_{p}^{\prime }$: softened version of student model, $y_{s}^{\prime }$: softened version of teacher model, $y_{h}$: the hard labels. α is a hyperparameter used to balance the two losses during training and β is calculated from α by the equation β = 1 − α.

## 4 METHODOLOGY

**FIGURE 2:**
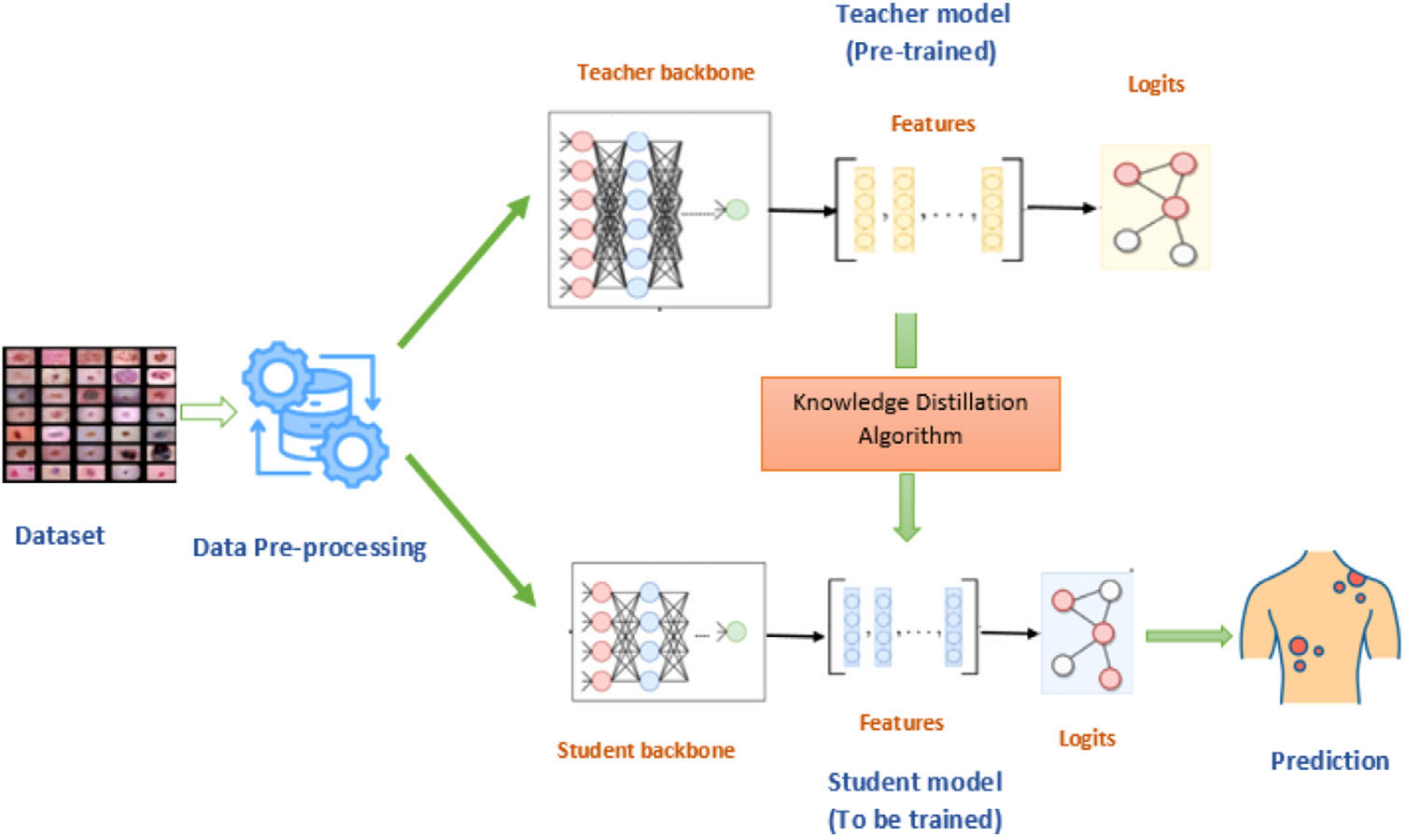
Proposed approach for classifying skin cancer on lightweight deep learning model.

The proposed approach encompasses two primary components: teacher modelling and student modelling. The teacher model is a substantial deep neural network use for training the ultimate student network. Conversely, the student network's purpose is to emulate a sizable ‘teacher’ model and harness the teacher's knowledge to achieve comparable or even superior levels of accuracy. However, in the following section, the proposed architecture is explained in detail in Figure 2.

### 4.1 Dataset

This experiment used the HAM10000 dataset [24], also known as Skin Cancer MNIST, which is popular in dermatology and machine learning and the ISIC 2019 dataset [25], which is extensively utilized in the study and development of computer‐aided diagnosis tools for skin cancer. The HAM10000 dataset consists of 10,015 dermatoscopic images, with diagnosis provided by a demographically diverse set of experts: 45% female, 55% male. The dataset includes seven different classes: actinic keratoses and intraepithelial carcinoma (Akiec, 327), basal cell carcinoma (Bcc, 514), benign keratosis‐like lesions (Bkl, 1,099), dermatofibroma (Df, 115), melanoma (Mel, 1,113), melanocytic nevi (Nv, 6,705), and vascular lesions (Vasc, 142). The ISIC2019 dataset contains 25,331 images across eight dermoscopic classes: AK, which is actinic keratosis with 867 images; BCC, basal cell carcinoma with 3323 images; BKL, benign keratosis with 2624; DF, dermatofibroma with 239; NV, melanocytic nevus with 12,875 images; MEL, melanoma with 4522 images; SCC, squamous cell carcinoma with 628 images; and VASC, vascular lesions with 253 images.

### 4.2 Data preprocessing

We began the data preprocessing phase by meticulously inspecting the training images. This visual representation provided a better understanding of the dataset, including its various categories or classes. We employed the ImageDataGenerator class, a powerful tool for increasing the diversity and variability of the training data. Several augmentation techniques have been applied, including rotation, shearing, zooming, brightness adjustment, and flipping.

Tables 2 and 3 show the number of images before and after augmentation for both HAM10000 and ISIC2019 dataset. If sufficient images were available, we select 1200 images; otherwise, we applied augmentation to the class to reach 1200 images. These techniques help improve the model's ability to generalize by providing additional variations in the images. To ensure consistent input for the machine learning model, the images are normalized using the rescale parameter of the ImageDataGenerator. This normalization step brings the pixel values within a constant range, typically between 0 and 1, facilitating model convergence and improved performance. We also set up data generators using the flow_from_directory() function. These data generators are crucial in efficient batch loading during model training. They automatically assign labels based on the directory structure, eliminating the need for manual label assignment. The generators are configured to yield batches of images and corresponding labels, enabling seamless integration with the model training process.

**TABLE 2:** Comparison of number of images for HAM10000 dataset before and after augmentation.

Class names	Before augmentation	After augmentation
Akiec	327	1200
Bcc	514	1200
Bkl	1099	1200
Df	1154	1200
Mel	1113	1200
Nv	6705	1200
Vasc	142	1200

**TABLE 3:** Comparison of number of images for ISIC 2019 dataset before and after augmentation.

Class names	Before augmentation	After augmentation
AK	867	1200
BCC	3323	1200
BKL	2624	1200
DF	239	1200
NV	12,875	1200
MEL	4522	1200
SCC	628	1200
VASC	253	1200

### 4.3 Teacher model

In the teacher modelling step, a large complex CNN model was trained for skin cancer classification. To train the teacher network, we used the ResNet 50, DenseNet 169, VGG16, and VGG19 architectures. All of these teacher models have an input size of 224 × 224 with three RGB channels. They used different numbers of convolution layers, max‐pooling layers, and fully connected layers followed by a softmax function for output. The convolutional layers used 3 × 3 filters with a stride of 1 and always used the same padding, and the pooling layers used 2 × 2 filters with a stride of 2. The preprocessed dataset was fitted to the teacher network, which was then trained on the target dataset. In this way, a network capable of extracting important attributes from the target dataset was formed. The model was prepared for knowledge distillation, enabling the transfer of knowledge from this network to any student network with a structure different from that of the teacher network. Table 4 shows the general structure and parameters of a sample small network.

**TABLE 4:** General structure and parameters of a sample small network architecture.

Type	Stride	Filter size	Input size
Convolution 1_1	1 × 1	3 × 3 × 3 × 8	224 × 224 × 3
Convolution 1_2	1 × 1	3 × 3 × 8 × 8	222 × 222 × 8
Convolution 2_1	1 × 1	3 × 3 × 8 × 16	110 × 110 × 8
Convolution 2_2	1 × 1	3 × 3 × 16 × 16	108 × 108 × 16
Convolution 3_1	1 × 1	3 × 3 × 16 × 32	53 × 53 × 16
Convolution 3_2	1 × 1	3 × 3 × 32 × 32	51 × 51 × 32
Convolution 4_1	1 × 1	3 × 3 × 32 × 64	24 × 24 × 32
Convolution 4_1	1 × 1	3 × 3 × 64 × 64	22 × 22 × 64
Convolution 5_1	1 × 1	3 × 3 × 64 × 64	10 × 10 × 64
Convolution 5_2	1 × 1	3 × 3 × 64 × 64	8 × 8 × 64

### 4.4 Student model

In the proposed system, we used a simple small network as a student model such that transferred information from a teacher model is fully utilized. This means the student model is designed as a simple network that leverages the teacher's knowledge to the fullest extent. The student model followed the same training methodology as the teacher model. The student model can also be adjusted using the hard labels of the datasets. A simple student model is described below: the input size of the student model is the same as that of the teacher model, that is, 224 × 224 with three RGB channels. The model also contains ten convolution layers, five max‐pooling layers, and three dense layers (fully connected layers). The convolutional layers used 3 × 3 with a stride of 1 and zero padding. The pooling layers were configured identically to those in the teacher model. However, the network was much smaller than the teacher model, requiring only 0.295 million parameters. A sample general structure and parameters for convolution layers of the student model are summarized in Table 4.

### 4.5 Knowledge distillation algorithm

#### 4.5.1 Related definitions

Definition 1 (Adam optimizer) Adam optimizer is a stochastic gradient descent method based on adaptive estimation for training deep learning networks. Generally, it has a faster calculation time and requires fewer parameters for tuning, therefore, makes it better than other optimization algorithms.

Definition 2 (Student loss) Student loss is the value of how much the student predictions differ from the ground truth using the softmax function.

Definition 3 (Distillation loss) Distillation loss is the value of how much soft student predictions are different from the soft teacher labels.

Definition 4 (Temperature) Temperature is a hyper‐parameter of the softmax function that is applied to transform the output logits into a probability distribution. The higher value of T will give us a softer probabilities distribution over the classes. On the other hand, a low temperature (less than 1) value makes the model more confident.

Definition 5 (Overall loss) The overall loss is composed of student loss and distillation loss which is calculated by the following equation:

OverallLoss=α×StudentLoss+β×DistillationLoss

where α and β are the coefficients. Generally, β = 1 − α.

#### 4.5.2 Classical knowledge distillation approach

**FIGURE 3:**
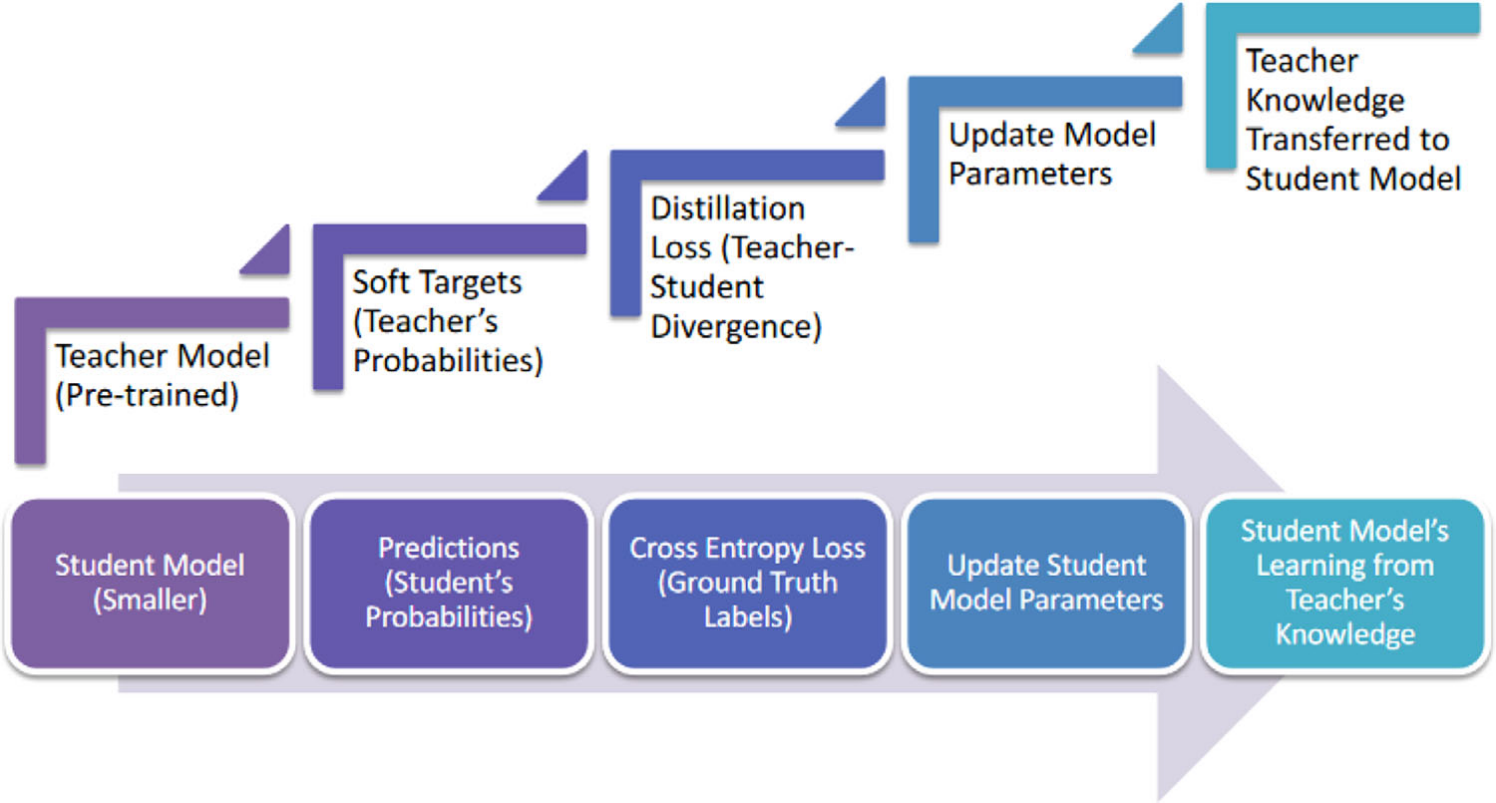
Classical knowledge distillation approach.

In Figure 3, we described the classical knowledge distillation algorithm where the teacher model is trained on a labelled dataset by optimizing an objective function to minimize the discrepancy between its predictions and the ground truth labels. Typically, the teacher model is larger and more accurate and is positioned in the upper part of the figure. On the other hand, the student model, which is smaller and being trained, is positioned at the lower part. The process begins with the teacher model generating soft targets (teacher's probabilities) based on its predictions. The student model then generates its own predictions (the student's probabilities) and compares them with the soft targets using the distillation loss (teacher student divergence). Simultaneously, the student model was trained using cross‐entropy loss with ground truth labels. The parameters of the student model were updated based on these losses, enabling it to learn from both the ground truth labels and the knowledge transferred by the teacher model. Through this process, the teacher's knowledge was effectively distilled and transferred to the student model.

#### 4.5.3 Proposed algorithm

The proposed approach aims to teach a smaller neural network, step by step, by leveraging the knowledge of a pre‐trained larger network. The pseudocode of the proposed module is presented in Algorithm 1 which outlines a structured process for iteratively training the smaller student network using soft target probabilities generated by the larger teacher network.

ALGORITHM 1 **Proposed method.**

**Table:** 

1:	**Input**: $T_{n}$ = A pre‐trained large teacher network; $S_{n}$ = An untrained small student network; $D_{r}$ = A large dataset; *B* = The batch size; O = An adaptive moment estimation optimizer; E = The number of epochs.
2:	**Output**: A trained small student network.
3:	**function** KnowledgeDistillationPseudocode $T_{n}$, $S_{n}$, $D_{r}$
4:	Initialize the teacher model ($T_{n}$).
5:	Split the dataset into $d_{1}$, $d_{2}$, …, $d_{m}$ disjoint sets where the size of each disjoint set is $D_{r}/B$.
6:	**for** i = 1 to E **do**
7:	**for** j = 1 to m **do**
8:	Draw the set $d_{j}$ of skin cancer classification images.
9:	Input the set $d_{j}$ into the teacher model.
10:	Generate soft target probabilities of the teacher for each sample in $d_{j}$.
11:	**end for**
12:	**end for**
13:	Initialize the student model ($S_{n}$).
14:	**for** i = 1 to E **do**
15:	knowledge_distillation_loss = 0
16:	**for** j = 1 to m **do**
17:	Retrieve the dataset $d_{j}$ of skin cancer classification images.
18:	Train the student model using the training dataset $d_{j}$ and teacher's soft targets.
19:	Compute the knowledge_distillation_loss.
20:	Modify the student model using the optimizer O.
21:	**end for**
22:	**if** **knowledge_distillation_loss does not decrease for E epochs** **then**
23:	**Terminate the execution of the program**.
24:	**else**
25:	**Continue**
26:	**end if**
27:	**end for**
28:	**end Function**

This module takes inputs of a well‐trained complex teacher model ($T_{n}$), an untrained basic student model ($S_{n}$), and a labelled dataset ($D_{r}$), systematically transforming the initially naive student network into a competent and compact one. The process starts with the initialization of the complex teacher model, followed by the division of the dataset into multiple disjoint subsets $d_{1}$, $d_{2}$, $d_{3}$, …., $d_{m}$ of equal size. The teacher model is trained on the labelled dataset for a specified number of epochs (E) while generating soft target probabilities for each subset (lines 6∼12). The student model ($S_{n}$) is initialized with random parameters at line 13 and is subsequently trained to mimic the teacher's knowledge over a series of training epochs. The training process was monitored for knowledge distillation loss, which was updated for each of the m batches of data from lines 14 to 18. Each batch involved drawing a subset of cancer classification images, inputting them into the student model, and training the student network based on the data and the teacher's soft targets. The knowledge distillation loss is computed to guide the student model's updates using an optimizer at line 19∼20. The algorithm monitored the loss, and if it failed to decrease over the specified number of epochs (E), the program was terminated. However, if the loss continues to decrease, training persists. Ultimately, the module produces a proficient and compact student network capable of emulating the teacher model's knowledge. This approach facilitates efficient knowledge transfer from the large teacher network to the smaller student network.

## 5 RESULTS AND DISCUSSION

In this section, we will start by describing our experimental setup. Next, we conduct experiments to highlight the importance of the proposed method. We explain the evaluation criteria and present a comparison of the results. Finally, the performance of the trained student models was evaluated.

### 5.1 Experimental setup

To conduct our experiment, we used Google Colab, a free GPU support cloud service that permits you to write and run Python code in your browser to build and train the deep learning models. Some libraries such as TensorFlow, Keras, NumPy and Matplotlib are used for inference in the Python simulation environment.

During the first phase of our experiment as shown in Table 5, we employed four reference teacher models: ResNet50, DenseNet169, VGG16, and VGG19. The design and the range of trainable parameters varied for each model. ResNet50 has a total of 24.5 million trainable parameters, followed by DenseNet169 with 13.3 million, VGG16 with 23.6 million, and VGG19 with 21 million. To facilitate training, we developed student models with reduced complexities. The first student model was a modified version of ResNet50 with 21 million trainable parameters. The second student model utilized DenseNet121 with 7.2 million trainable parameters. The third student model employed VGG16 with 16.8 million trainable parameters, and the fourth student model used VGG19 with 19 million trainable parameters. Throughout the training process, we fine‐tuned the settings and weights of the student models to enhance their performance for skin cancer classification. Our aim was to improve their accuracy of categorizing different types of skin cancer. In the next phase as shown in Table 6, we introduced a custom‐made student model with significantly reduced complexity, featuring only 0.29 million trainable parameters. Despite its compact architecture, this student model aimed to examine the impact of a smaller and more streamlined design on classification performance. We used the same teacher models for comparison and reference.

**TABLE 5:** Experimental models for phase 1.

Teacher model	Student model
ResNet‐50 (24.5M params)	ResNet‐50 (21M params)
DenseNet‐169 (13.3M params)	DenseNet‐121 (7.2M params)
VGG‐16 (23.6M params)	VGG‐16 (16.8M params)
VGG‐19 (21M params)	VGG‐19 (19M params)

**TABLE 6:** Experimental models for phase 2.

SL.	Teacher model	Student model
1.	Resnet 50 (24.5M trainable parameters)	Small network (0.29M trainable parameters)
2.	DenseNet169 (13.3M trainable parameters)	
3.	VGG 16 (23.6M trainable parameters)	
4.	VGG19 (21M trainable parameters)	

In knowledge distillation for this case, the parameter reduction is achieved by architectural simplification where the student model is designed with fewer layers or neurons than the large teacher model and training it to mimic the teacher's performance by learning from ‘soft targets'—probability distributions produced by the teacher. These soft targets reveal important patterns and class relationships, allowing the student to focus on essential features without requiring the teacher's full complexity. Using a loss function that combines real labels with soft targets, the student learns to replicate the teacher's knowledge efficiently. This approach allows the student to perform similarly to the teacher with far fewer parameters. Tables 5 and 6 show the number of parameters in the teacher models and in student model after parameter reductions in both phases 1 and 2.

During the simulation, we evaluated the performance of our models on the HAM10000 (10K images) and ISIC2019(34K Images) datasets. Among them, 20% of the images were randomly chosen for the test set and validation, while the rest of the images were chosen for the training set. Before fitting the images into the deep CNN models, we uniformly reshaped all images to 224 × 224 and performed data augmentation using common image processing operations with horizontal and vertical flips, luminance adjustments, contrast, saturation, scaling, etc. The teacher model was pre‐trained and fine‐tuned for ten epochs using an Adam optimizer with an initial learning rate of 0.0001, controls the moving average, beta (β) of 0.9, and decay of 0.0001. Early stopping approach was also used every ten consecutive epochs if the model performance was not enhanced.

### 5.2 Performance of experimental models for phase 1

**FIGURE 4:**
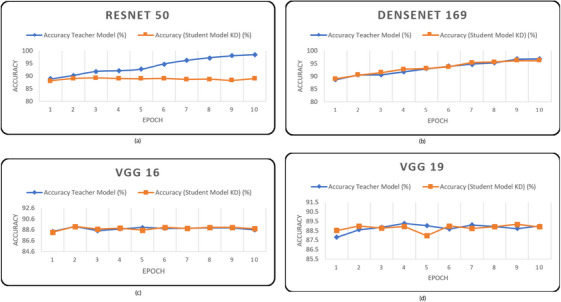
Accuracy for phase 1 based on teacher model (a) ResNet50, (b) DenseNet161, (c) VGG16 and (d) VGG19 on HAM10000 dataset.

We initially evaluated the performance of our models on the HAM10000 dataset. Upon reviewing the results, we observed that the teacher models achieved higher average accuracy compared to the student models, indicating their superior classification performance, as shown in Table 7 and Figure 4.

**TABLE 7:** Accuracy in phase 1 based on HAM10000 dataset.

Model	Average accuracy (Teacher)	Average accuracy (Student)	Average accuracy (Student with KD)
ResNet50	94.82%	81.61%	88.69%
DenseNet169	93.30%	88.72%	93.24%
VGG16	89.21%	88.87%	89.05%
VGG19	89.00%	88.73%	88.89%

From Table 7 and Figure 4, it can be said that the student models with knowledge distillation showed promising results, often approaching the performance of the teacher models. On the HAM10000 dataset, the teacher model ResNet50 exhibited the highest average accuracy of 94.82%, while the student model with knowledge distillation achieved 88.69% accuracy and the student model without knowledge distillation achieved 81.61% accuracy. Similarly, DenseNet169 demonstrated strong performance, achieving an average accuracy of 93.30% as the teacher model, 88.72% without knowledge distillation, and 93.24% with knowledge distillation. In contrast, VGG16 delivered results with an average accuracy of 89.21% as the teacher model, 88.87% without knowledge distillation, and 89.05% with knowledge distillation. As for VGG19, it achieved an average accuracy of 89.00% as the teacher model, 88.73% without knowledge distillation, and 88.89% with knowledge distillation. When evaluating the models on the ISIC2019 dataset, we observed slightly lower overall accuracies as shown in Table 8. For DenseNet169, the student model with knowledge distillation achieved a slightly higher average accuracy of 84.13% compared to the teacher model's accuracy of 83.70%. However, the student model without knowledge distillation had a slightly lower accuracy of 81.76%. This indicates that the knowledge distillation process provided a small boost in the performance of the student model. For VGG19, both the student model without knowledge distillation and the student model with knowledge distillation obtained similar average accuracies of 82.14%. The teacher model had a slightly higher accuracy of 82.86%. In this instance, knowledge distillation had a negligible effect on the student model's performance in comparison to the instructor model. The capability and complexity of the models and the unique properties of the datasets used are potential explanations for the results. On the other hand, applying the knowledge distillation approach to ResNet50 and VGG16 models yielded the anticipated outcomes: the student model trained with knowledge distillation achieved higher accuracy than the student model trained without knowledge distillation but still falls short of matching the accuracy of the teacher model. DenseNet169 is a more sophisticated and parameter‐rich model compared to VGG19, which could explain why the student model with knowledge distillation benefited more in terms of accuracy improvement. Additionally, the data distribution and the difficulty of the classification task might have varied, leading to differing impacts of knowledge distillation. Furthermore, it is important to note that the impact of knowledge distillation can vary depending on numerous factors such as the architecture of the models, the size of the datasets, and the specific optimization techniques used during training. Additionally, the optimal implementation of knowledge distillation requires careful tuning of the hyperparameters and balancing between the teacher and student models. From Table 9, we can see that RestNet‐50 achieves 94.63% precision, 94.51% recall and 94.13% F1‐score for the teacher model while student model without distillation achieves 81.72% precision, 81.48% recall and 81.71% F1‐score which is lower than that of the teacher model. The student model with knowledge distillation achieved better performance than that without distillation, with a precision of 88.64%, recall of 88.57%, and an F1‐score of 88.55%. DenseNet‐169, VGG‐16, and VGG‐19 also demonstrated good performance as student models, both with and without distillation, on the HAM10000 dataset. However, the teacher model consistently outperformed the models. On the other hand, from Table 10, it is evident that all student models perform similarly to their teacher model on the ISIC‐2019 dataset in terms of precision, recall, and F1‐score.

**TABLE 8:** Accuracy in phase 1 based on ISIC2019 dataset.

Model	Average accuracy (Teacher)	Average accuracy (Student)	Average accuracy (Student with KD)
ResNet50	82.30%	81.61%	82.14%
DenseNet169	83.70%	81.76%	84.13%
VGG16	82.31%	81.96%	82.16%
VGG19	82.86%	82.14%	82.14%

**TABLE 9:** Performance metrics in phase 1 for different models on HAM10000 dataset.

	Precision			Recall			F1‐Score		
Model	Teacher	Student	Student (KD)	Teacher	Student	Student (KD)	Teacher	Student	Student (KD)
ResNet‐50	94.63	81.72	88.64	94.51	81.48	88.57	94.13	81.71	88.55
DenseNet169	93.96	88.68	93.26	93.89	88.94	93.19	93.61	88.63	93.48
VGG‐16	89.86	88.34	89.36	89.81	88.61	89.37	89.64	88.43	89.22
VGG‐19	89.67	88.58	88.61	89.64	88.71	88.96	89.84	88.91	88.98

**TABLE 10:** Performance metrics in phase 1 for different models on ISIC‐2019 dataset.

	Precision			Recall			F1‐Score		
Model	Teacher	Student	Student (KD)	Teacher	Student	Student (KD)	Teacher	Student	Student (KD)
ResNet‐50	82.57	81.62	81.93	82.80	81.65	82.63	82.75	81.23	82.24
DenseNet169	83.17	81.24	84.12	83.64	81.52	84.43	83.12	81.51	84.32
VGG‐16	82.42	81.14	82.20	82.89	81.46	82.33	82.71	81.65	82.00
VGG‐19	82.42	82.36	82.97	82.27	82.51	82.79	82.00	82.36	82.88

A paired *t*‐test was performed to show that the accuracy levels for both the teacher and student models, which were both trained using the HAM10000 and ISIC2019 datasets, were statistically different. The mean accuracy for the teacher models was higher for the HAM10000 dataset; however, it did not reach statistical significance, *t* = 1.51, *p* = 0.228, making the performance difference negligible. Here, the *t*‐value quantifies the observed difference, while the *p*‐value determines whether this difference is significant. On the ISIC2019 dataset, however, the difference, though from higher accuracy by the teacher models, did not reach a level of statistical significance but was near, with t = 2.65, p = 0.077 below the conventional threshold of 0.05. These findings indicate that minor differences in accuracy could exist, but the success achieved by the student models significantly reduced complexity and further strengthened the success of knowledge distillation in resource‐efficient deployments. This reinforces the idea that, in real‐world scenarios, a student model is highly relevant, reassuring its validity in settings where computational efficiency and model simplicity are required with no loss of diagnostic performance.

### 5.3 Performance of experimental models for phase 2

**FIGURE 5:**
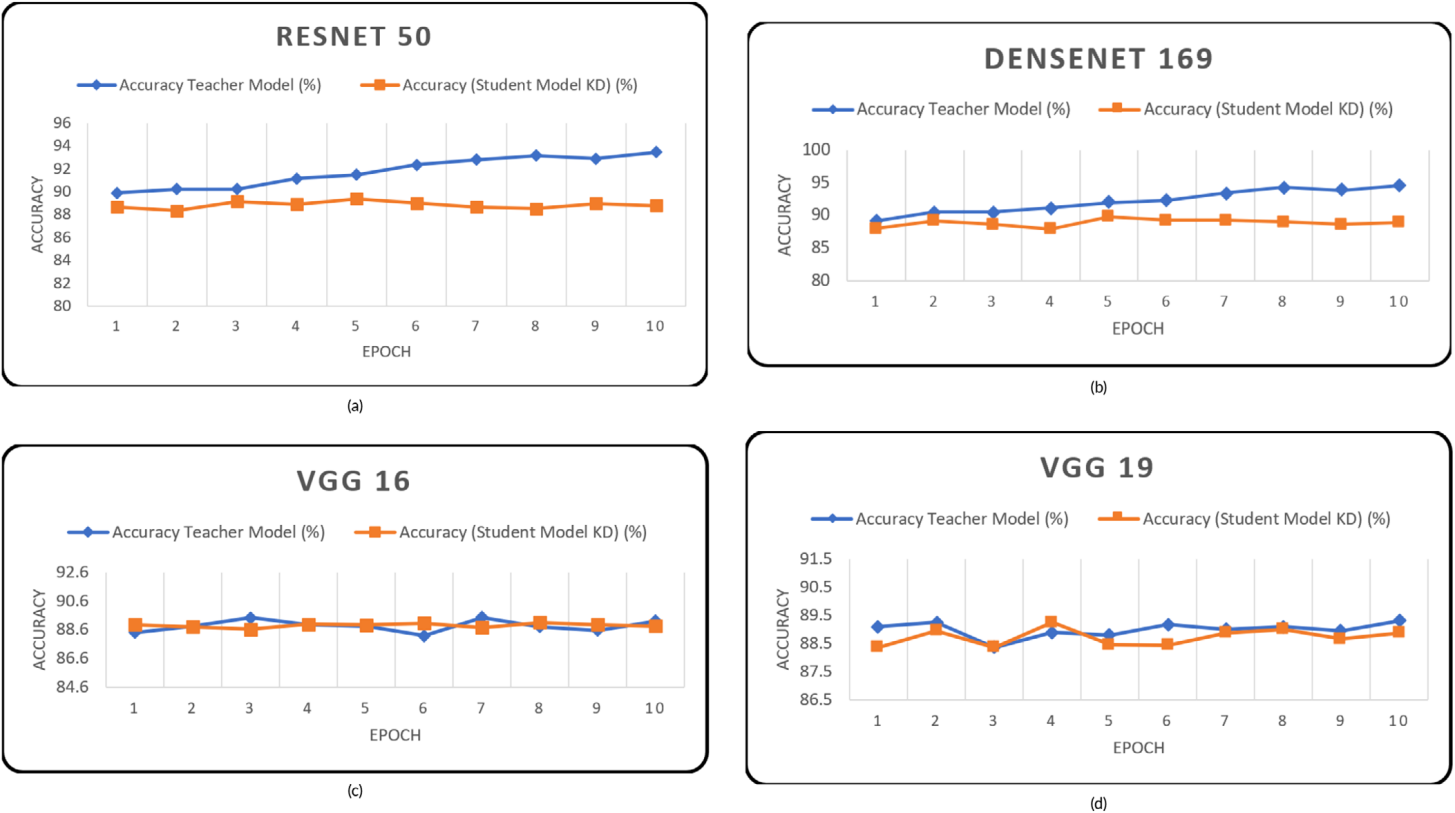
Accuracy of phase 2 based on teacher model (a) ResNet50, (b) DenseNet161, (c) VGG16 and (d) VGG19 on HAM10000 dataset.

In phase 2 of the experiment, when knowledge distillation was used, the results showed differences in the average accuracy between the instructor models and custom‐made student model with significantly reduced complexity as shown in Figure 5 and Table 11. The same teacher model from phase 1 was used here.

**TABLE 11:** Accuracy based on HAM10000 in phase 2.

Model	Average accuracy (Teacher)	Average accuracy (Student with KD)
ResNet50	94.82%	88.99%
DenseNet169	93.30%	88.96%
VGG16	89.21%	88.86%
VGG19	89.00%	88.63%

As from the Table 11 and Figure 5, the student model with knowledge distillation achieved an average accuracy of 88.99% for ResNet50, compared to the teacher model's 94.82% accuracy on the HAM10000 dataset. Similarly, with DenseNet‐169, the teacher model attained an average accuracy of 93.30%, while the student model with knowledge distillation achieved 88.96% accuracy. For VGG16, the student model with knowledge distillation showed a modest improvement, reaching 88.86%, compared to the teacher model's 89.21%. Finally, for VGG19, the student model with knowledge distillation achieved an average accuracy of 88.63%, while the teacher model reached 89%.

As from the Table 12, the student model with knowledge distillation achieved an average accuracy of 81.82% for ResNet50, which was close to the teacher model's average accuracy of 82.30%. With DenseNet‐169, the teacher model achieved an average accuracy of 83.70%, and the student model with knowledge distillation reached 83.42%. Similarly, for VGG16, the student model achieved 82.18%, nearly matching the teacher model's 82.31%. For VGG19, the student model attained 81.39%, which was slightly lower than the teacher's model of 82.86%.

**TABLE 12:** Average accuracy based on ISIC‐2019 in phase 2.

Model	Average accuracy (Teacher)	Average accuracy (Student with KD)
ResNet50	82.30%	81.82%
DenseNet169	83.70%	83.42%
VGG16	82.31%	82.18%
VGG19	82.86%	81.39%

From Table 13, it can be observed that the ResNet50 student model with knowledge distillation achieved a precision of 88.14%, recall of 88.31%, and an F1‐score of 88.74% on the HAM10000 dataset, which are slightly lower than those of the teacher model. The same teacher model from phase 1 was used in phase 2. Similarly, DenseNet‐169, VGG‐16, and VGG‐19 performed well as student models on the HAM10000 dataset, showing competitive results despite being slightly outperformed by their respective teacher models. On the other hand, as shown in Table 14, all teacher and student models with knowledge distillation demonstrated similar performance in terms of precision, recall, and F1‐score on the ISIC‐2019 dataset in phase 2.

**TABLE 13:** Precision, recall, and F1‐score based on HAM10000 in phase 2.

Model	Precision (Teacher)	Precision (Student with KD)	Recall (Teacher)	Recall (Student with KD)	F1‐Score (Teacher)	F1‐Score (Student with KD)
ResNet50	94.63	88.14	94.51	88.31	94.13	88.74
DenseNet169	93.96	88.34	93.89	88.29	93.61	88.61
VGG16	89.86	88.25	89.81	88.26	89.64	88.04
VGG19	89.67	88.29	89.64	88.38	89.84	88.68

**TABLE 14:** Precision, recall, and F1‐score based on ISIC‐2019 in phase 2.

Model	Precision (Teacher)	Precision (Student with KD)	Recall (Teacher)	Recall (Student with KD)	F1‐Score (Teacher)	F1‐Score (Student with KD)
ResNet50	82.57	81.94	82.80	81.86	82.75	82.00
DenseNet169	83.17	83.47	83.64	83.51	83.12	83.57
VGG16	82.42	82.13	82.89	82.22	82.71	82.26
VGG19	82.42	81.45	82.27	81.41	82.00	81.43

These results are presented for a follow‐up experiment to explore the extent to which knowledge distillation improves the performance of different students compared to their teachers. Indeed, all teacher models ResNet50, DenseNet169, VGG16, and VGG19 recorded higher baseline accuracies compared to their student models using knowledge distillation; in fact, these were marginal improvements since accuracy reductions generally fell within a range of −2%. Similar to phase 1, we also performed a paired t‐test in order to have a more sophisticated measure of the differences in performance accuracies between teacher models and their knowledge‐distillation‐enhanced student counterparts. Using the accuracy results in Tables 11 and 12, the significance level indicators (p‐values) for the two datasets, HAM10000 and ISIC2019, are 0.147 and 0.146, respectively. This indicates that the test did not find statistically significant differences in the observed performances. Thus, the student model with knowledge distillation performs similarly to the teacher model. This result also suggests that knowledge distillation is a promising approach for achieving high performance with simpler models.

## 6 CONCLUSION

This study introduced an efficient approach for skin cancer classification using knowledge distillation techniques. It involves transferring knowledge from a larger, well‐trained deep neural network to a smaller, more lightweight network to advance the accuracy of model while retaining its runtime efficiency. The performance of the lightweight model and the larger teacher model in classifying skin cancer disease was evaluated using publicly available datasets. The presented model greatly improved the prediction ability of the lightweight model, producing a more efficient and precise solution for skin cancer detection. In addition, this study opens up opportunities to create highly effective and precise deep‐learning models for various medical imaging applications. Overall, this research provides the results of more resource‐efficient and accurate models for skin cancer detection, which can assist in the early diagnosis and treatment of life‐threatening skin cancer. This approach seeks to provide a more resource‐efficient and accurate solution for skin cancer detection.

## AUTHOR CONTRIBUTIONS

**Suman Saha**: Conceptualization; investigation; methodology; supervision; validation; writing—original draft; writing—review and editing. **Md. Moniruzzaman Hemal**: Data curation; investigation; methodology; validation; visualization; writing—original draft. **Md. Zunead Abedin Eidmum**: Investigation; methodology; validation; visualization; writing—review and editing. **Muhammad Firoz Mridha**: Formal analysis; investigation; validation; writing—review and editing.

## CONFLICT OF INTEREST STATEMENT

The authors declare no conflicts of interest.

## Data Availability

Dataset that supports the findings of this research is openly available on ISIC‐2019 Challenge's website at https://challenge.isic‐archive.com/data/ and HAM10000 Dataset at https://doi.org/10.7910/DVN/DBW86T.
